# Single Physical Performance Measures Cannot Identify Geriatric Outpatients with Sarcopenia

**DOI:** 10.14283/jfa.2018.19

**Published:** 2018-08-01

**Authors:** S.M.L.M. Looijaard, S.J. Oudbier, E.M. Reijnierse, G.J. Blauw, C.G.M. Meskers, A.B. Maier

**Affiliations:** 1 Department of Internal Medicine, Section of Gerontology and Geriatrics, VU University Medical Center, Boelelaan 1117, 1081 HV, Amsterdam, The Netherlands; 2 Department of Medicine and Aged Care, @AgeMelbourne, The Royal Melbourne Hospital, University of Melbourne, 300 Grattan Street, Parkville, 3050, Melbourne, Victoria, Australia; 3 Department of Gerontology and Geriatrics, Leiden University Medical Center, Albinusdreef 2, 2333 ZA, Leiden, The Netherlands; 4 Department of Geriatrics, Bronovo Hospital, Bronovolaan 5, 2597 AX, The Hague, The Netherlands; 5 Department of Rehabilitation Medicine, VU University Medical Center, Boelelaan 1117, 1081 HV, Amsterdam, The Netherlands; 6 Department of Human Movement Sciences, @AgeAmsterdam, Faculty of Behavioral and Movement Sciences, Vrije Universiteit Amsterdam, Amsterdam Movement Sciences, van der Boechorststraat 7, 1081 BT, Amsterdam, The Netherlands

**Keywords:** Aged, geriatrics, physical performance, sarcopenia

## Abstract

**Background:**

Sarcopenia is highly prevalent in the older population and is associated with several adverse health outcomes. Equipment to measure muscle mass and muscle strength to diagnose sarcopenia is often unavailable in clinical practice due to the related expenses while an easy physical performance measure to identify individuals who could potentially have sarcopenia is lacking.

**Objectives:**

This study aimed to assess the association between physical performance measures and definitions of sarcopenia in a clinically relevant population of geriatric outpatients.

**Design, setting and participants:**

A cross-sectional study was conducted, consisting of 140 community-dwelling older adults that were referred to a geriatric outpatient clinic. No exclusion criteria were applied.

**Measurements:**

Physical performance measures included balance tests (sideby- side, semi-tandem and tandem test with eyes open and -closed), four-meter walk test, timed up and go test, chair stand test, handgrip strength and two subjective questions on mobility. Direct segmental multi-frequency bioelectrical impedance analysis was used to measure muscle mass. Five commonly used definitions of sarcopenia were applied. Diagnostic accuracy was determined by sensitivity, specificity and area under the curve.

**Results:**

Physical performance measures, i.e. side-by-side test, tandem test, chair stand test and handgrip strength, were associated with at least one definition of sarcopenia. Diagnostic accuracy of these physical performance measures was poor.

**Conclusions:**

Single physical performance measures could not identify older individuals with sarcopenia, according to five different definitions of sarcopenia.

## Introduction

Prevalence rates of sarcopenia, defined as age-related low muscle mass and muscle strength, vary between 0% and 15% in healthy older individuals and between 2% and 34% in geriatric outpatients, depending on the applied definition (1). Sarcopenia is associated with decreased mobility, a higher risk of falls, dependency in activities of daily living, morbidity and mortality (2, 3, 4). Although there is no consensus yet on the definition of sarcopenia, the majority of definitions contain a measure of muscle mass and/or muscle strength (5, 6, 7, 8, 9, 10).

Equipment to measure muscle mass and muscle strength is often unavailable in clinical practice due to the related expenses (11). Poor physical performance has been shown to be associated with sarcopenia (12, 13, 14). Recently, a prediction model was proposed to identify sarcopenia with the use of demographic parameters and physical performance measures (15). However, such a prediction model is time consuming due to its inclusion of multiple physical tests and complex calculations. Identifying individuals who could potentially have sarcopenia in clinical practice would be greatly facilitated when an easy to use physical performance measure could be applied to identify individuals with sarcopenia. This could lead to the identification of individuals who could potentially have sarcopenia, who could then be referred to diagnose sarcopenia.

This study aims to assess the association between physical performance measures and different definitions of sarcopenia in a clinically relevant population of geriatric outpatients.

## Methods

### Study design

This cross-sectional study consisted of 140 community-dwelling older adults who were referred to a geriatric outpatient clinic of a middle-sized teaching hospital (Bronovo, The Hague, The Netherlands) between March 2011 and January 2012. These older adults were referred because of mobility problems for a comprehensive geriatric assessment. The study originally consists of 299 older adults, but muscle mass measurements were only available in 140 older adults as these measurements were later added as part of clinical care. No exclusion criteria were applied; inclusion was based on referral. The study was approved by the Medical Ethical Committee of the Leiden University Medical Center (Leiden, the Netherlands). Informed consent was waived as this study was based on regular care.

### Study population characteristics

Medical charts were used to retrieve information on age, sex and medical history. Medical history included information on the presence of: hypertension, myocardial infarction, chronic obstructive pulmonary disease, diabetes mellitus, rheumatoid- or osteoarthritis, Parkinson's disease and malignancy. The presence of two or more of these diseases was defined as multimorbidity. Anthropometric measurements included height and body weight and were measured to the nearest 0.1 decimal. Cognitive functioning was measured by the Mini Mental State Examination (MMSE) resulting in a score ranging from 0 to 30 points, higher scores indicating better cognitive function (16).

### Physical performance measures, objective

Physical performance measures included balance tests, fourmeter walk test, timed up and go (TUG), chair stand test (CST) and handgrip strength (HGS).

Balance tests were performed in three different positions (side-by-side, semi-tandem and tandem) according to the protocol of the Short Physical Performance Battery (17), and were performed with eyes open and with eyes closed. Individuals were classified as unable to maintain for ten seconds (0) and able to maintain for ten seconds (1). Tandem balance test with eyes closed was excluded from the present analysis as the number of individuals who were able to maintain in the tandem position for ten seconds was less than five individuals.

Gait speed was obtained by a four-meter walk test where individuals were asked to walk at their usual pace (17). The best performance of two measurements was used and expressed in meters per second.

The TUG measures the time in seconds needed to stand up from a sitting position without using hands, walk three meters, walk around a cone, walk three meters back and return to sitting position without using hands, as fast as possible.

The CST measures the time in seconds needed to stand up five times from sitting position to a straight standing position and sit down again while keeping the arms crossed over the chest, as fast as possible (17).

HGS was measured using a hydraulic handheld dynamometer (Jamar, Sammons Preston, Inc., Bolingbrook, IL, USA). Individuals were asked to squeeze as hard as possible three times with the right and left hand side alternately. Maximal HGS of the three trials was used for analysis (18) and expressed in kilograms.

Higher gait speed and HGS implied a better physical performance while a higher TUG and CST time implied a lower physical performance. For all physical performance measures, all individuals who could not perform or finish the test or used hands to stand up from a sitting position, were given a time of 100 seconds.

### Physical performance measures, subjective

In addition to the objective physical performance measures, two questions were asked: 1) Falls: “Did you fall in the past year?” (yes/no) and 2) Difficulty with walking: “Do you experience difficulty with walking?” (yes/no).

### Sarcopenia definitions

Muscle mass was measured using direct segmental multi-frequency Bioelectrical Impedance Analysis (DSM-BIA; InBody 720, Biospace Co., Ltd, Seoul, Korea) (19). Five definitions of sarcopenia were used to examine the association between physical measures and sarcopenia: 1) Baumgartner et al. using appendicular lean mass (ALM) divided by height2 (5); 2) Janssen et al. using relative skeletal muscle mass (SM) (SM divided by body mass) (6); 3) European Working Group on Sarcopenia in Older Persons (EWGSOP) using an algorithm of gait speed, HGS and skeletal muscle index (SMI; SM divided by height2) (7); 4) Foundation for National Institutes of Health (FNIH) definition one using HGS and ALM divided by body mass index (BMI) (8) and 5) The International Working Group on Sarcopenia (IWGS) using gait speed and ALM divided by height2 (9).

### Statistical analysis

Continuous variables were reported by mean ± standard deviation (SD) if data was normally distributed or median [interquartile range (IQR)] for skewed distributions. Associations between physical performance measures (independent variables) with definitions of sarcopenia (dependent variables) were analyzed with binary logistic regression analysis. Two models were used: the crude model and an adjusted model for sex and age. P-values of less than 0.05 were considered statistically significant.

For the statistically significant associations between physical performance measures and definitions of sarcopenia, sensitivity, specificity and the area under the curve (AUC) were calculated to determine the diagnostic accuracy. Sensitivity and specificity were defined as low <70%, moderate 70–90% and high >90%. AUC was defined as low <0.70, acceptable 0.70–0.80, excellent 0.80–0.90 and outstanding >0.90. To test diagnostic accuracy, CST and HGS were dichotomized: CST ≥13 seconds (14, 20), and HGS <20 kilograms for females and <30 kilograms for males were considered low (21). Statistical analyses were performed using Statistical Package for Social Sciences (SPSS Inc, Chicago, USA), version 22.

## Results

Table 1 shows the characteristics of the geriatric outpatients, with a mean age of 80.9 (7.1) years and 42% males. Table 2 shows the applied definitions of sarcopenia and the prevalence of sarcopenia. Prevalence of sarcopenia ranged from 3.6% to 23.6%, depending on the definition.

**Table 1:** Characteristics of geriatric outpatients (N=140)

	N	Total
General characteristics		
Sex, male	140	59 (42.1)
Age, years, mean ± SD	140	80.9 ± 7.08
Body weight, kg, mean ± SD	140	71.9 ± 15.2
Height, cm, mean ± SD	140	167.2 ± 9.93
Health characteristics		
Multimorbidity	134	55 (41.0)
MMSE, median [IQR]	140	27 [25–29]
Physical performance measures, objective		
Side-by-side eyes open, able to maintain	140	135 (96.4)
Semi-tandem eyes open, able to maintain	140	120 (85.7)
Tandem eyes open, able to maintain	140	53 (37.9)
Side-by-side eyes closed, able to maintain	138	114 (82.6)
Semi-tandem eyes closed, able to maintain	138	69 (50.0)
Tandem eyes closed, able to maintain	138	3 (2.2)
Gait speed, m/s, mean ± SD	140	0.77 ± 0.27
TUG, sec, median [IQR]	135	16.6 [12.1–26.2]
CST, sec, median [IQR]	138	15.6 [11.9–26.5]
HGS, kg, mean ± SD, males	59	33.1 ± 6.01
HGS, kg, mean ± SD, females	81	21.3 ± 5.11
Physical performance measures, subjective		
History of falling last 12 months, yes	140	89 (63.6)
Difficulty walking last 12 months, yes	140	101 (72.1)
Muscle mass parameters		
ALM/height2, kg/m2, mean ± SD	140	7.16 ± 1.21
ALM/BMI, mean ± SD	140	0.80 ± 0.21
SMI, kg/m2, mean ± SD	140	9.21 ± 1.31
SM/body mass, %, mean ± SD	140	64.0 ± 8.79


All results are given in number (percentage) unless indicated otherwise. SD: standard deviation; MMSE: Mini Mental State Examination, score 0–30; IQR: interquartile range; TUG: Timed Up and Go; CST: Chair Stand Test; HGS: Handgrip Strength; ALM: Appendicular Lean Mass; BMI: Body Mass Index; SMI: Skeletal Muscle Index; SM: Skeletal Muscle.

**Table 2:** Prevalence of sarcopenia in geriatric outpatients according to the applied definitions of sarcopenia

Definition	Diagnostic criteria	Cutoff point		Prevalence, n (%)
		Males	Females	
Baumgartner (1998)	ALM/height2	≤7.26 kg/m2	≤5.45 kg/m2	28 (20.0)
Janssen (2002)	SM/body weight x 100%	<37%	<28%	24 (17.1)
EWGSOP (2010)	1. Gait speed	≤0.8 m/s	≤0.8 m/s	33 (23.6)
	SMI (SM/height2)	≤10.75 kg/m2	≤6.75 kg/m2	
	2. Gait speed	>0.8 m/s	>0.8 m/s	
	HGS	<30 kg	<20 kg	
	SMI (SM/height2)	≤10.75 kg/m2	≤6.75 kg/m2	
IWGS (2011)	Gait speed	<1.0 m/s	<1.0 m/s	28 (20.0)
	ALM/height2	≤7.23 kg/m2	≤5.67 kg/m2	
FNIH (2014)	HGS	<26 kg	<16 kg	5 (3.6)
	ALM/BMI	<0.789	<0.512	


ALM: Appendicular Lean Mass; SM: Skeletal Muscle; EWGSOP: European Working Group on Sarcopenia in Older People; HGS: Handgrip Strength; SMI: Skeletal Muscle Index; IWGS: International Working Group on Sarcopenia; FNIH: Foundation for the National Institutes of Health; BMI: Body Mass Index.

Table 3 shows the association between physical performance measures and sarcopenia according to the applied definitions. Out of all balance tests, the ability to perform the tandem stance with eyes open was most often associated with a decreased likelihood to have sarcopenia (Baumgartner et al., EWGSOP and IWGS). CST was associated with sarcopenia by use of the EWGSOP and IWGS definitions. HGS was associated with sarcopenia using the definition of Baumgartner et al. and IWGS. The other physical performance measures i.e. semi-tandem balance test with eyes open and eyes closed, gait speed, TUG and the subjective physical performance measures did not show an association with any of the definitions of sarcopenia.

**Table 3:** Physical performance measures and sarcopenia according to the applied definitions

	Baumgartner		Janssen		EWGSOP		IWGS		FNIH	
	OR	95% CI	OR	95% CI	OR	95% CI	OR	95% CI	OR	95% CI
Side-by-side, eyes open*	1.00	0.11–9.31	0.82	0.09–7.69	1.24	0.13–11.5	0.36	0.06–2.25	0.12	0.01–1.36
Adjusted model	1.47	0.15–14.4	0.60	0.06–6.46	1.32	0.10–17.5	0.58	0.09–3.85	0.05	0.00–0.82
Semi-tandem, eyes open*	1.49	0.41–5.49	0.80	0.24–2.65	0.51	0.19–1.42	1.00	0.31–3.27	0.66	0.07–6.18
Adjusted model	1.74	0.46–6.65	0.61	0.17–2.18	0.25	0.06–1.02	1.25	0.37–4.26	0.45	0.04–4.70
Tandem, eyes open*	0.29	0.10–0.82	1.83	0.75–4.44	0.44	0.18–1.07	0.29	0.10–0.82	1.10	0.18–6.80
Adjusted model	0.33	0.11–1.00	1.68	0.60–4.42	0.26	0.08–0.81	0.38	0.13–1.14	0.65	0.08–5.30
Side-by-side, eyes closed*	0.52	0.19–1.41	0.76	0.25–2.29	0.23	0.09–0.57	0.43	0.16–1.13	0.84	0.09–7.83
Adjusted model	0.58	0.21–1.65	0.66	0.21–2.10	0.07	0.01–0.33	0.51	0.18–1.40	0.66	0.07–6.58
Semi-tandem, eyes closed*	0.76	0.33–1.77	0.82	0.34–1.98	0.67	0.30–1.48	0.48	0.20–1.13	1.52	0.25–9.41
Adjusted model	0.94	0.38–2.34	0.62	0.23–1.66	0.51	0.19–1.39	0.63	0.25–1.59	1.09	0.16–7.74
Gait speed, m/s	0.41	0.08–2.07	1.54	0.30–7.83	NA	NA	NA	NA	0.72	0.02–21.4
Adjusted model	0.44	0.08–2.46	0.79	0.14–4.48	NA	NA	NA	NA	0.30	0.01–11.2
TUG, sec	1.01	1.00–1.02	1.00	0.99–1.01	1.00	0.99–1.02	1.01	1.00–1.02	1.00–1.02	0.99–1.04
Adjusted model	1.01	1.00–1.02	1.00	0.99–1.02	1.00	0.99–1.02	1.01	1.00–1.02	1.02	0.99–1.04
CST, sec	1.01	1.00–1.02	1.00	0.99–1.01	1.01	0.99–1.02	1.01	1.00–1.02	1.01	0.99–1.03
Adjusted model	1.01	1.00–1.02	1.00	0.99–1.08	1.02	1.00–1.03	1.01	1.00–1.02	1.02	0.99–1.04
HGS, kg	0.97	0.92–1.02	1.03	0.98–1.09	NA	NA	0.96	0.91–1.01	NA	NA
Adjusted model	0.86	0.78–0.94	0.94	0.87–1.03	NA	NA	0.85	0.77–0.94	NA	NA
Difficulty walking, yes	0.77	0.32–1.89	2.16	0.69–6.79	1.04	0.43–2.49	1.20	0.47–3.10	1.57	0.17–14.5
Adjusted model	0.68	0.27–1.71	2.34	074–7.44	1.96	0.70–5.47	1.16	0.43–3.15	1.72	0.18–16.0
History of falling, yes	1.27	0.53–3.06	0.95	0.38–2.35	1.00	0.45–2.26	1.56	0.63–3.85	2.35	0.26–21.6
Adjusted model	1.25	0.51–3.04	0.94	0.38–2.33	1.29	0.50–3.32	1.61	0.63–4.09	2.25	0.24–21.0


OR: Odds Ratio; CI: Confidence interval; EWGSOP: European Working Group on Sarcopenia in Older People; IWGS: International Working Group on Sarcopenia; FNIH: Foundation for the National Institutes of Health; NA: Not Applicable; TUG: Timed Up and Go; CST: Chair Stand Test; HGS: Handgrip Strength. Adjusted model adjusted for sex and age. All results with a p-value < 0.05 are considered significant and are given in bold; *Balance tests were dichotomized into unable to maintain for ten seconds (0) and able to maintain for ten seconds (1).

Table 4 shows the diagnostic accuracy for physical performance measures significantly associated with sarcopenia. The tandem balance test with eyes open showed moderate sensitivity and low specificity and AUC for all three sarcopenia definitions. The side-by-side test with eyes closed showed low sensitivity, moderate specificity and low AUC for the EWGSOP definition. CST showed low sensitivity, specificity and AUC for the EWGSOP and IWGS definitions. HGS showed low sensitivity, moderate specificity and low AUC for the Baumgartner et al. and IWGS definitions.

**Table 4:** Diagnostic accuracy of physical performance measures according to the applied definitions of sarcopenia

		Baumgartner	Janssen	EWGSOP	IWGS	FNIH
Side-by-side, eyes open*	Sensitivity	NA	NA	NA	NA	20.0
	Specificity	NA	NA	NA	NA	97.0
	AUC	NA	NA	NA	NA	0.59 [0.30–0.87]
Tandem, eyes open*	Sensitivity	82.1	NA	75.8	82.1	NA
	Specificity	42.9	NA	42.1	42.9	NA
	AUC	0.63 [0.52–0.73]	NA	0.59 [0.48–0.70]	0.63 [0.52–0.73]	NA
Side-by-side, eyes closed*	Sensitivity	NA	NA	36.4	NA	NA
	Specificity	NA	NA	88.6	NA	NA
	AUC	NA	NA	0.63 [0.51–0.74]	NA	NA
CST, dichotomized	Sensitivity	NA	NA	81.8	85.7	NA
	Specificity	NA	NA	34.3	34.5	NA
	AUC	NA	NA	0.42 [0.31–0.53]	0.40 [0.29–0.51]	NA
HGS, dichotomized	Sensitivity	50.0	NA	NA	53.6	NA
	Specificity	75.0	NA	NA	75.9	NA
	AUC	0.63 [0.50–0.75]	NA	NA	0.65 [0.53–0.77]	NA


Sensitivity and specificity are given in percentage; AUC is given with 95% confidence interval. EWGSOP: European Working Group on Sarcopenia in Older People; IWGS: International Working Group on Sarcopenia; FNIH: Foundation for the National Institutes of Health; NA: Not Applicable; AUC: Area under the curve; CST: Chair stand test; HGS: Handgrip strength. NA indicates non-significant results in logistic regression analyses for which no diagnostic accuracy was calculated. All results with a p-value < 0.05 are considered significant and are given in bold. *Balance tests were dichotomized into unable to maintain for ten seconds (0) and able to maintain for ten seconds (1).

## Discussion

Physical performance measures, i.e. side-by-side test, tandem test, CST and HGS, were associated with sarcopenia using several definitions, but diagnostic accuracy was poor.

Previous studies have shown that tandem balance test, gait speed, CST and HGS are valid and reliable measures to assess physical performance (22, 23). Moreover, gait speed, CST and HGS have proven to be associated with sarcopenia according to the EWGSOP definition in community dwelling older adults (20, 24, 25). Unfortunately, the results of this study in geriatric outpatients did not show a single suitable physical performance measure to identify older individuals with sarcopenia. For a test to be suitable to identify individuals with a high risk on sarcopenia, it needs to have a high sensitivity as especially false-negatives are undesirable. Sensitivity was low to moderate, which would mean that many individuals who could potentially have sarcopenia would be missed. Moreover, specificity and AUC were low and therefore single physical performance measures have poor diagnostic accuracy to identify individuals with sarcopenia.

Sarcopenia is associated with negative health outcomes and therefore an important diagnosis in clinical practice. Nutritional and physical interventions have proven to increase muscle mass, muscle strength and physical performance (26, 27). Improving physical performance measures might reduce the risk of sarcopenia and therewith its negative health outcomes. Early recognition of sarcopenia is necessary to initiate interventions. BMI is a measurement that is often used to identify individuals who are at risk of adverse health outcomes, however, BMI does not encompass the risk of sarcopenia (28) Another proposed screening tool to identify individuals with sarcopenia is the SARC-F, a simple five-item questionnaire (subjective measures of strength, assistance in walking, rise from a chair, climb stairs, falls). This is one of the screening instruments that is increasingly being used in community-dwelling middle-aged to older adults to identify individuals who could potentially have sarcopenia (29, 30).

Multimorbidity was high in this population of geriatric outpatients and this could be an explanation for the lack of diagnostic accuracy of physical performance measures to identify older individuals with sarcopenia because physical performance could also be inflicted by disease-specific mechanisms.

To the best of our knowledge, this is the first study outlining the association between various objective physical performance measures and several commonly used definitions of sarcopenia in a clinically relevant population of geriatric outpatients. Validated physical performance measures were used. Furthermore, the population is heterogeneous and no exclusion criteria were used which makes it a good representation of the actual older population visiting outpatient clinics. A limitation of this study could be that only BIA and not Dual Energy X-ray Absorptiometry (DEXA) was used to determine muscle mass parameters of sarcopenia. However, BIA and DEXA showed high agreement in a population of community-dwelling individuals (19).

Single physical performance measures could not identify older individuals with sarcopenia, according to five different definitions. Therefore, no easy and fast method to identify individuals with sarcopenia can be recommended. Future research should focus on developing and validating screening tools so that individuals with a high probability of having sarcopenia can be identified. Individuals who are considered to be at risk of sarcopenia should be referred to diagnose sarcopenia using the diagnostic criteria that are used in the definitions of sarcopenia.

*Acknowledgements:* We thank M. Stijntjes and J.H. Pasma for their contribution to the study.

*Funding:* This study was supported by the Dutch Technology Foundation STW, which is part of the Netherlands Organization for Scientific Research (NWO) and which is partly funded by the Ministry of Economic Affairs. Furthermore, this study was supported by the seventh framework program MYOAGE (HEALTH-2007-2.4.5-10) and 050-060-810 Netherlands Consortium for Healthy Aging (NCHA). The funders had no role in study design, data collection and analysis, decision to publish, or preparation of the manuscript.

*Statement of authorship:* All authors have made substantial contributions to all of the following: 1) conception and design of the study, acquisition of data or analysis and interpretation of data; 2) drafting the article or revising it critically for important intellectual content; 3) final approval of the version to be submitted.

*Conflict of Interest:* S.M.L.M. Looijaard: none to declare. S.J. Oudbier: none to declare. E.M. Reijnierse: none to declare. G.J. Blauw: none to declare. C.G.M. Meskers: none to declare. A.B. Maier: none to declare.
